# Supplementary material for: MBD8 is required for LDL2-mediated transcriptional repression downstream of H3K9me2 in Arabidopsis
Source: Nucleic Acids Res. 2026 May 11;54(9):gkag361. doi: 10.1093/nar/gkag361 (PMC13157976; doi:10.1093/nar/gkag361)
Supplement: gkag361_Supplemental_Files [file gkag361_supplemental_files.zip › Legend of Supplementary Tables.docx]

Supplementary Table S1

List of oligonucleotides used for the MBD8–DNA binding assay

Supplementary Table S2

Summary of proteins detected by IP-MS
