# Supplementary material for: MBD8 is required for LDL2-mediated transcriptional repression downstream of H3K9me2 in Arabidopsis
Source: Nucleic Acids Res. 2026 May 11;54(9):gkag361. doi: 10.1093/nar/gkag361 (PMC13157976; doi:10.1093/nar/gkag361)
Supplement: gkag361_Supplemental_Files [file gkag361_supplemental_files.zip › Supplementary_Figures.pdf]

Supplementary Figure S1

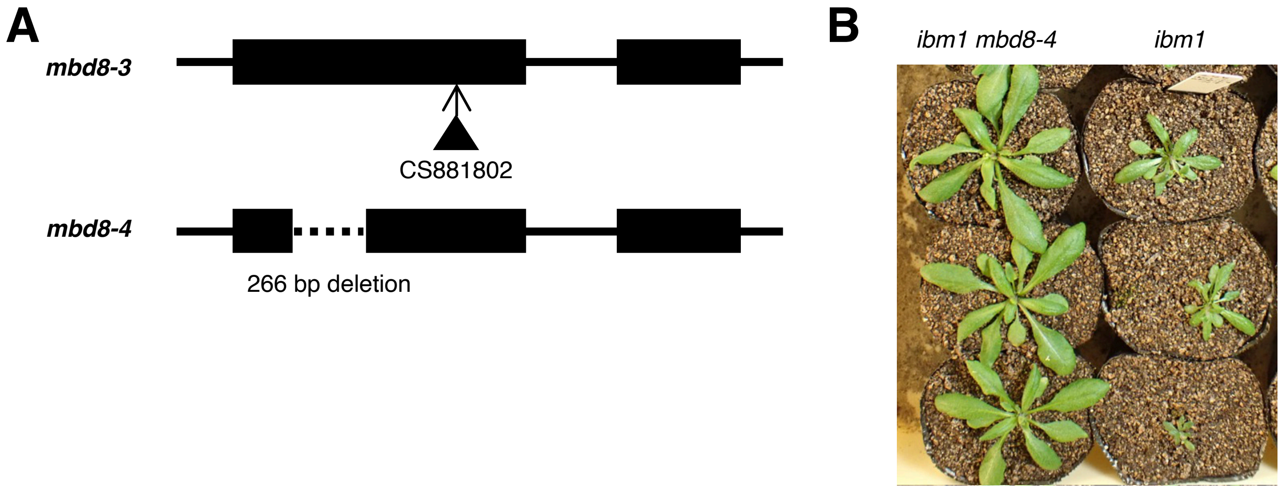

Supplementary Figure S1. *mbd8* mutants' alleles and *mbd8* mutants' phenotype.

A. Graphical representation of the *MBD8* gene showing the position of the mutations. The black boxes represent the exons. **B.** Phenotype of *ibm1* and *ibm1 mbd8-4* mutants.

Supplementary Figure S2

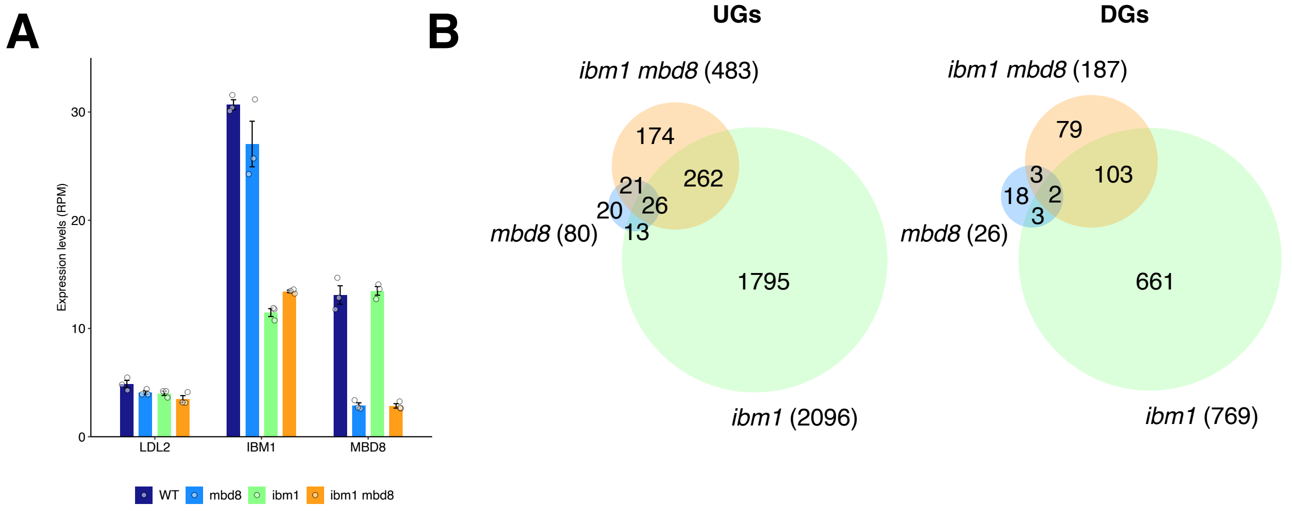

C

mbd8

UGs	protein coding gene	57
	pseudo gene	1
	TE	7
	non-coding RNA	11
	novel transcribed region	4
		80
DGs	protein coding gene	22
	pseudo gene	0
	TE	1
	non-coding RNA	2
	novel transcribed region	1
		26

ibm1

UGs	protein coding gene	1916
	pseudo gene	18
	TE	50
	non-coding RNA	101
	novel transcribed region	11
		2096
DGs	protein coding gene	701
	pseudo gene	11
	TE	17
	non-coding RNA	38
	novel transcribed region	2
		769

ibm1 mbd8

UGs	protein coding gene	387
	pseudo gene	10
	TE	42
	non-coding RNA	33
	novel transcribed region	11
		483
DGs	protein coding gene	163
	pseudo gene	4
	TE	6
	non-coding RNA	11
	novel transcribed region	3
		187

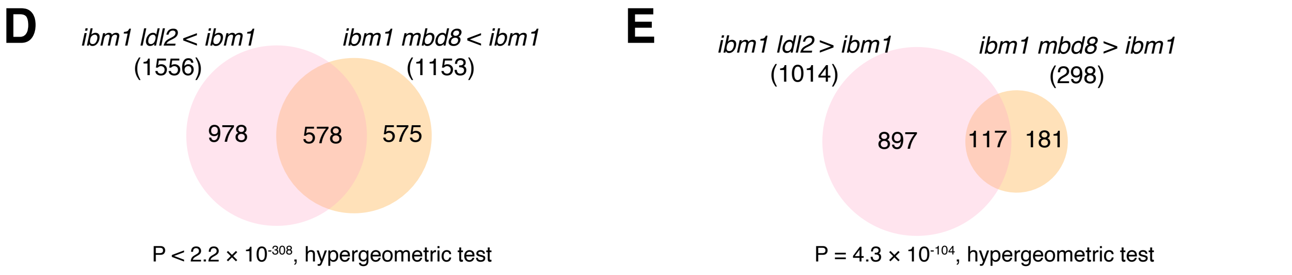

Supplementary Figure S2. mRNA-seq analysis in each mutant.

A. mRNA level (reads per million mapped reads; RPM) of *LDL2*, *IBM1* and *MBD8* genes in WT, *mbd8*, *ibm1*, and *ibm1 mbd8*. Means and SD for three biological replicates are shown. **B.** Venn diagram of the up-regulated genes (UGs) and downregulated genes (DGs) in each. **C.** The numbers of up-regulated and down-regulated genes in each mutant. **D.** Ven diagram representation of the significant overlaps between down-regulated genes in *ibm1 ldl2* compared to *ibm1* (35) and down-regulated genes in *ibm1 mbd8* compared to *ibm1* (P <2.2×10^{-308}, hypergeometric test). **E.** Ven diagram representation of the significant overlaps between up-regulated genes in *ibm1 ldl2* compared to *ibm1* (35) and up-regulated genes in *ibm1 mbd8* compared to *ibm1* (P = 4.3×10^{-104}, hypergeometric test).

Supplementary Figure S3

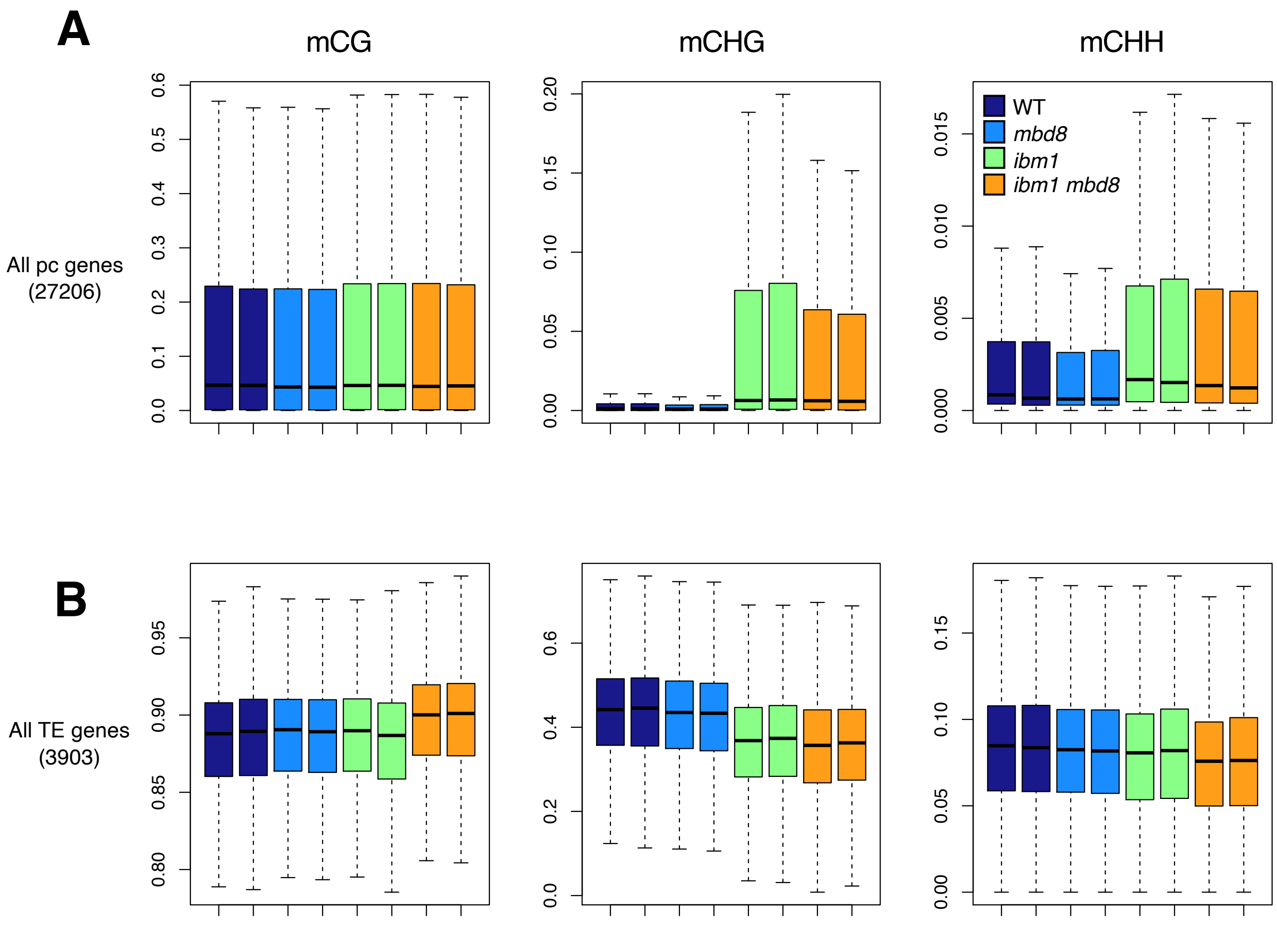

Supplementary Figure S3. Whole-genome DNA methylation analysis in each mutant.

A, B. Boxplots showing DNA methylation levels in each context (CG, CHG, and CHH; H can be A, T, or C) in all protein-coding genes (**A**), all TE genes (**B**). Two biological replicates are shown for each genotype.

Supplementary Figure S4

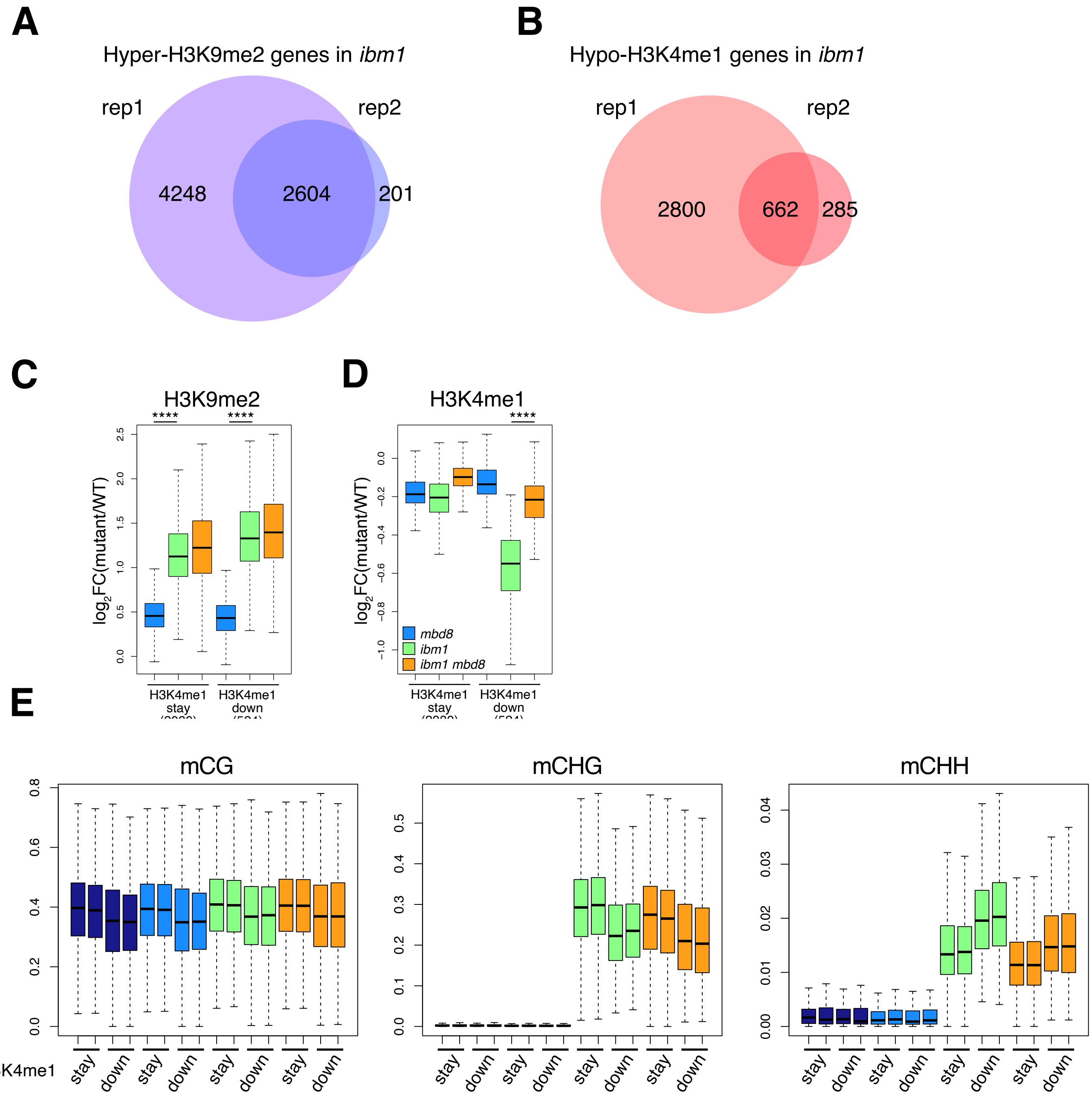

Supplementary Figure S4. Hyper-H3K9me2 genes and Hypo-H3K4me1 genes in *ibm1*.

A. The overlap of genes with increased H3K9me2 in *ibm1* compared to WT (H3K9me2 RPKM change (*ibm*1–WT) > 2) between two biological replicates. "Hyper-H3K9me2 genes in *ibm1*" are defined as the overlapping genes (n=2,604). **B.** The overlap of genes with decreased H3K4me1 in *ibm1* compared to WT (H3K4me1 RPKM change (*ibm*1–WT) < -2) between two biological replicates. "Hypo-H3K4me1 genes in *ibm1*" are defined as the overlapping genes (n=662). **C, D.** A biological replicate of the experiment shown Fig. 2C, D. Boxplots showing changes in H3K9me2 levels (**C**) and changes in H3K4me1 (**D**). Y-axis represents $\log_2$ fold change in RPKM values (mutant/WT). H3K4me1-stay genes (n=2,080) and H3K4me1-down genes (n=524) were analyzed, excluding genes shorter than 1.0 kb. ****P < 0.0001, Wilcoxon test. **E.** Boxplots showing DNA methylation levels in each context (CG, CHG, and CHH) in H3K4me1-stay genes, and H3K4me1-down genes. Two biological replicates are shown for each genotype.

Supplementary Figure S5

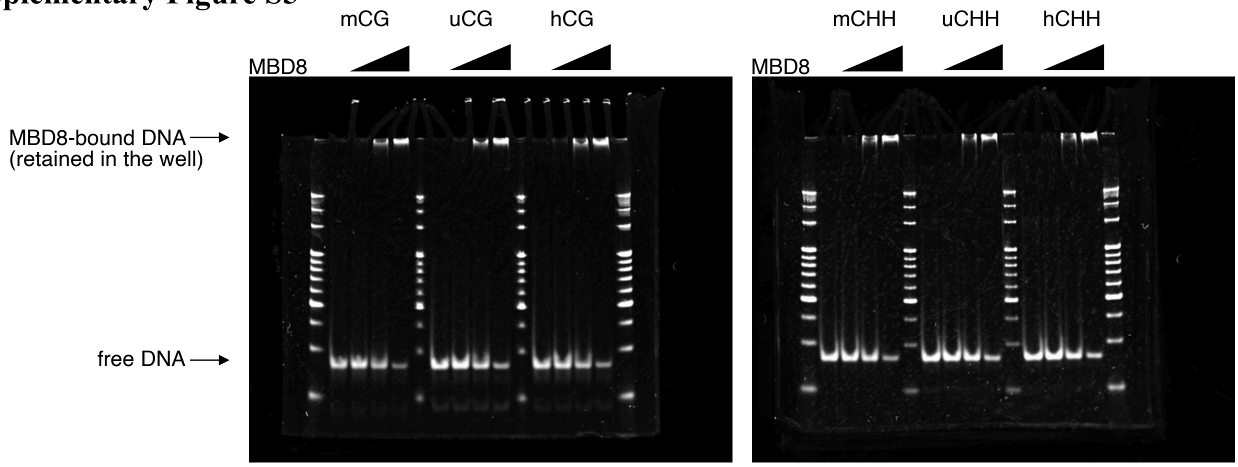

Supplementary Figure S5. EMSA for DNA binding activity of MBD8 to CG and CHH sites.

EMSA showing the binding of MBD8 to DNA that contains CG and CHH sites. We used oligonucleotides that were fully methylated (m), unmethylated (u), and hemimethylated (h).

Supplementary Figure S6

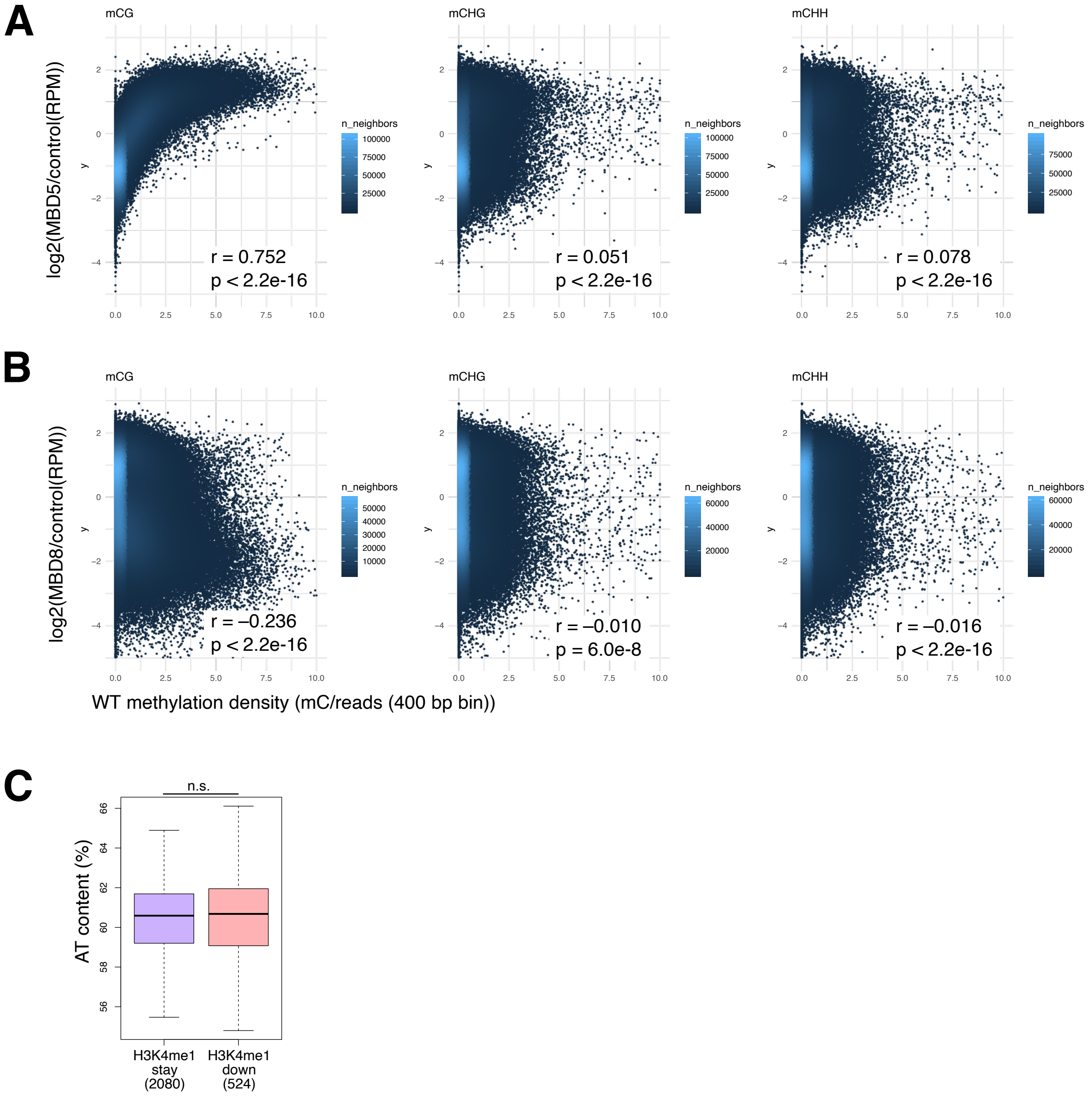

Supplementary Figure S6. Binding specificity of MBD8.

A, B. Scatterplots showing the correlation of MBD5 DAP-seq signals (**A**) or MBD8 DAP-seq signals (**B**) and DNA methylation density (mC are normalised to mapped reads) in the indicated context. Each dot represents a 400 bp bin. **C.** Boxplots showing AT content in H3K4me1-stay genes (n=2,080) and H3K4me1-down genes (n=524). Wicoxon test. n.s., not significant.

Supplementary Figure S7

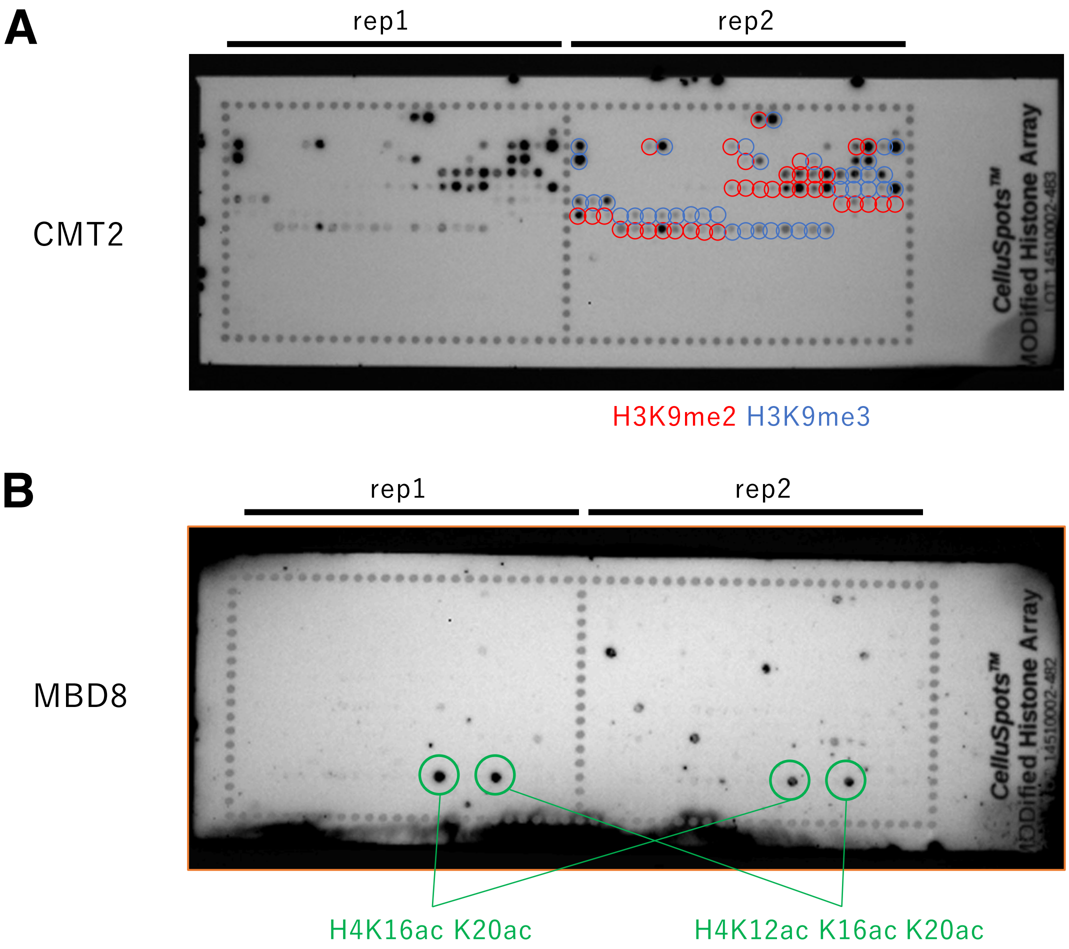

Supplementary Figure S7. Histone peptide array.

A. CMT2 binding assay using a histone peptide array. Red and blue circles indicate peptides containing di-, and trimethylated H3K9 peptides, respectively. **B.** MBD8 binding assay. Peptides containing both H4K16ac and H4K20ac (green circles) are reproducibly bound to MBD8.

Supplementary Figure S8

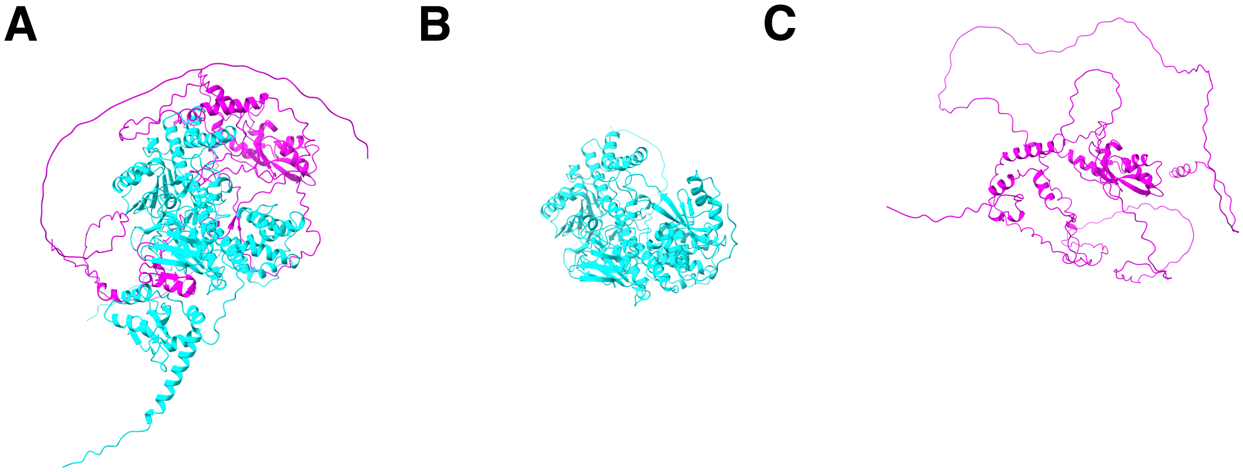

Supplementary Figure S8. MBD8 and LDL2 structure predicted by Alphafold2.

A. MBD8-LDL2 complex structure predicted by Alphafold2. This is the same as (**Fig. 4C**). **B.** LDL2 monomer structure. **C.** MBD8 monomer structure.

Supplementary Figure S9

```
                                         ****
LDL3 (875-934) SDVSYVSDVSAMDNSKHKVRVSTSNGCEYLGDAVLVTVPLGCLKAETIKFSPPLPDWKYA
 FLD (400-449) QTIRYGSNGVK----------VTAGNQVYEGDMVLCTVPLGVLKNGSIKFVPELPQRKLD
LDL1 (484-533) ESIRYGSNGVL----------VYTGNKEFHCDMALCTVPLGVLKKGSIEFYPELPHKKKE
LDL2 (375-424) DTIKYGDGGVE----------VISGSQIFQADMILCTVPLGVLKKRSIKFEPELPRRKQA
```

Supplementary Figure S9. Amino acid alignment of LDL2 and its paralogs.

In the alignment, positions conserved or highly similar among the proteins are colored in green for hydrophobic amino acids (VILM), blue for basic (KRH) amino acids, and pink for acidic (DE) amino acids. The asterisk symbol (*) refers to amino acid residues required for binding to MBD8. Protein names and domain limits (in parentheses) are indicated at the start of the sequences.

Supplementary Figure S10

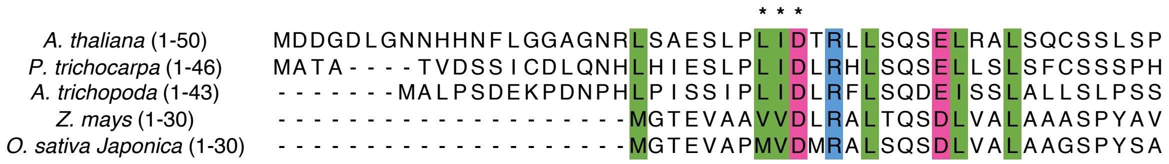

Supplementary Figure S10. Amino acid alignment of the N-terminal region of MBD8 and its orthologs.

In the alignment, positions conserved or highly similar among the MBD8 proteins are colored in green for hydrophobic amino acids (VILM), blue for basic (KRH) amino acids, and pink for acidic (DE) amino acids. The asterisk symbol (*) refers to amino acid residues required for binding to LDL2. Host species and domain limits (in parentheses) are indicated at the start of the sequences. NCBI accession numbers are as follows: *Arabidopsis thaliana*: NP_173650.3, *Popilus trichocarpa*: KAI5597816.1, *Amborella trichopoda*: XP_020528307.1, *Zea mays*: NP_001123600.1, *Oryza sativa japonica*: XP_ 015611640.1.

Supplementary Figure S11

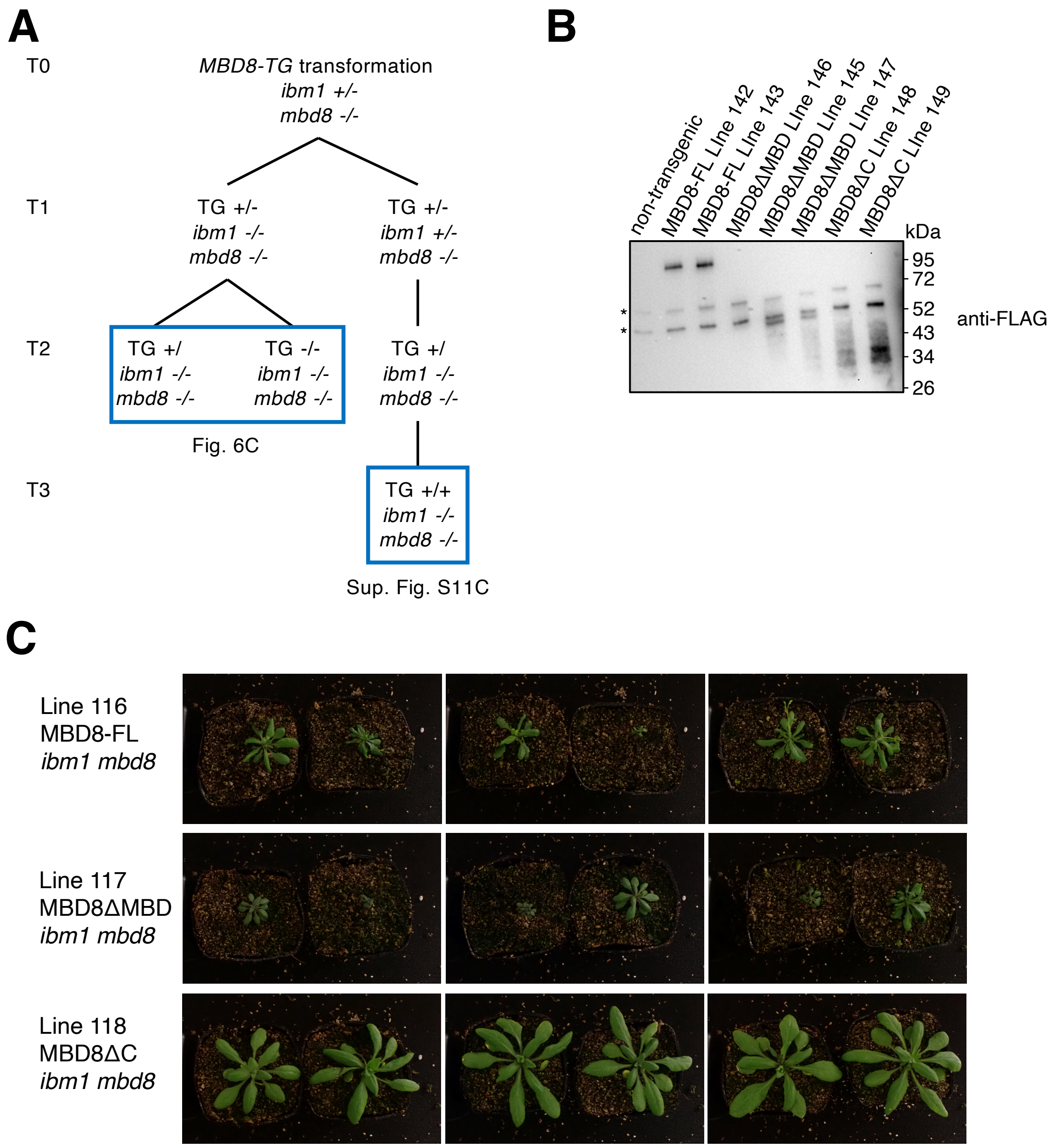

Supplementary Figure S11. MBD8 complementation test.

A. Scheme of the MBD8 complementation test. Phenotypes of the plants marked with blue outlines were observed. **B.** Western blot analysis showing the expression of MBD8 transgenes blotted with anti-FLAG. The asterisks indicate non-specific bands.**C.** Phenotypes of WT, *ibm1*, *ibm1 mbd8*, and each T3 transgenic line. MBD8-FL, MBD8ΔMBD, and MBD8ΔC constructs were transformed into the *ibm1 mbd8* background.
